# Adolescent Health: A Framework for Developing an Innovative Personalized Well-Being Index

**DOI:** 10.3389/fped.2020.00181

**Published:** 2020-05-07

**Authors:** Francesca Mastorci, Luca Bastiani, Cristina Doveri, Gabriele Trivellini, Anselmo Casu, Cristina Vassalle, Alessandro Pingitore

**Affiliations:** ^1^Clinical Physiology Institute, CNR, Pisa, Italy; ^2^Fondazione G. Monasterio, Regione Toscana, Pisa, Italy

**Keywords:** adolescence, health, personalized well-being, prevention, school, emotion, social context

## Abstract

Adolescence is not only typically considered a time of good health but also characterized by an emergence of risk factors that may have long-term consequences for well-being that represents strong predictors of adverse health outcomes. The aim of the study is to assess adolescence well-being through the development of an integrated Well-Being Index (WBI) including variables of lifestyle habits, social context, emotional status, and mental skills. One thousand two hundred forty-eight healthy adolescents (Female 48%; Male 52%; mean age 13 years) were recruited from five Italian junior high schools, by KIDSCREEN-52 and cognitive processing using the Stroop Test. School performance was estimated by questions concerning the scholastic achievement. Social context was the most important predictor of perceived well-being (β = 0.972, SE = 0.014, *p* < 0.0001), with parent relation (*p* < 0.0001) as the most observed variable. Subsequently, WBI was strongly represented by lifestyle habits (β = 0.954, SE = 0.017, *p* < 0.000) with autonomy (*p* < 0.0001), and emotional status (β = 0.949, SE = 0.017, *p* < 0.000) with psychological well-being perception (*p* < 0.0001). Finally, mental skills (β= −0.1417, SE = 0.031, *p* < 0.0.00) was the least important predictor for the WBI index (*p* < 0.0001). Personalised (P) WBI was obtained by the sum of each centered and scaled WBI variable, weighted by the corresponding ratio between factor loading and residual variance. Social context was the more important predictor of WBI, followed by lifestyle, emotional factors, and lastly mental skills. PWBI provides an integrated and personalized perspective of adolescents' well-being, on the basis of a cooperation between school, family, and community with the common intent to promote and protect adolescent health.

## Introduction

In the last decades, we assisted to a substantial change from intervention to promotion and prevention, in line with the new concept of health, considered as a continuum from absence of disease to a well-being condition (1). According to this new perspective, the improvement of health and well-being during adolescence is a topic of growing interest in several fields, including public policy, school programs, and preventive medicine. Adolescence is defined as the transitional period from childhood to adulthood, characterized by critical physical, psychological, emotional, and social changes (2). Moreover, adolescence is a period of risk taking, and unhealthy behaviors often do not only affect health within this period but also later in life. Moreover, adolescence is considered a period of contradictions; in fact, at the same time, it is the healthiest period of the entire lifespan (with respect to psychophysical parameters) and the time in which one-third of the total disease burden of adulthood is determined (3). Interestingly, growing evidences indicate that adolescence is a dynamic and flexible period of knowledge and adaptation to target health interventions, so that adolescents can make positive lifestyle choices to enhance their well-being (4, 5). In this view, strategies, including health-promoting skills, positive behaviors, and social connection with family, school, and community are recommended in order to limit the incidence of a health-jeopardizing conduct. Usually, health and well-being evaluation instruments, as expressed by existing personal well-being index, include life satisfaction and the subjective psychological assessment of mood happiness (6). However, to our knowledge, it is not yet available a tool for health and well-being monitoring that, according to a multiparametric approach, includes different health aspects, such as lifestyle habits, emotional status, social context, and mental skills.

Therefore, the aim of this study was to assess adolescence well-being through the development of an integrated index taking into account several variables of lifestyle habits, social context, emotional status, and mental skills in a school-based study of 1,659 adolescents.

## Methods

### Study Participants

Data in the AVATAR study were collected between 2017 and 2018. Ten junior high schools participated in the AVATAR project, acronym for “A new purpose for promotion and eVAluation of healTh and well-being Among healthy teenageRs.” AVATAR project is aimed to develop a new tool to assess lifestyle habits, social context, emotional status, and mental skills in adolescents, and to define an integrated index of the best indicators of well-being (7). In total, 1,659 boys and girls, aged between 10 and 14 years, were included. Adolescent students were enrolled according to the following inclusion criteria: age 10–14 years, absence of neuropsychiatric or other diseases, informed consent signed, and filling of the entire questionnaires proposed. Of 1,659, 411 were excluded for the following reasons: diagnosed neuropsychiatric or other diseases (*n* = 57), absence of sign informed consent (*n* = 169), and questionnaires not filled out completely (*n* = 185). Therefore, the final population consisted of 1,248 adolescents (Female 48%; Male 52%; mean age 12 ± 0.81 yrs). In every school class, all the adolescents filled out the questionnaire, and whether they were not eligible due to exclusion criteria reasons, they were excluded from the study retrospectively.

Participants were previously instructed on how to fill out the questionnaires and conduct the tests. All tests were performed during participants' computer lesson in school time. No incentive was provided to adolescents or parents. A research assistant was available to provide information and technical support to complete questionnaires. The study was approved by the internal ethics committee of each participating school, in accordance with the Italian law. In addition, all parents or legal guardians gave informed consent, and authorized researchers to use their data in accordance with the Italian law. All procedures performed in the study were in accordance with the ethical standards of the institutional and/or national research committee and with the 1964 Helsinki declaration and its later amendments or comparable ethical standards.

### Data Collection

Data were collected with AVATAR Web-tool (7). A sociodemographic data record was used to collect information on gender, age, schooling, family structure, and body mass index, according to WHO age group (8). The Italian version of KIDSCREEN-52 was used to assess health-related quality of life (9, 10). The KIDSCREEN is a self-report questionnaire designed to address health-related quality of life, aimed to monitor, and measure the personal experiences in children and adolescent about their perception of health status and well-being. The questionnaire, that describes physical, psychological, mental, social, and functional aspects of well-being, consists of 52 items grouped in 10 dimensions (9, 10). KIDSCREEN questionnaires is psychometrically tested using data obtained in a multicenter European study which included a sample of 22,827 children recruited in 13 countries (11). Dietary habits were evaluated using the Mediterranean Diet Quality Index for children and adolescents (KIDMED) (12). The KIDMED index was based on principles sustaining Mediterranean dietary patterns as well as those that undermine it. The index ranged from 0 to 12, and consisted of a self-administered 16-question test. Physical activity levels were assessed using the Physical Activity Questionnaire for Older Children (PAQ-C). The questionnaire provides a general measure of physical activity for 8–20-year olds. The PAQ-C is a self-administrated questionnaire consisting of nine items rated on a five-point scale. A higher score indicates more active children/adolescents (13).

The cognitive processing was estimated with the Stroop Color and Word Test Children's Version (14). This test, standardized for the identification of executive function deficits in children and adolescents, evaluates the Stroop performance as measure of ability for planning, directing and maintaining attention, organization, abstract reasoning and problem-solving, self-regulation, and motor control.

The school engagement has been estimated through questions concerning the scholastic achievement in language and literature, language acquisition, and science.

### Avatar Methodology

The proposed multifactorial approach is focused on the integration of four components of health-related well-being [lifestyle habits (LH); emotional status (ES); social context (SC); and mental skills (MS)], as perceived by adolescents, in order to create the Well-Being Index (WBI) in the first step and Personalised Well-Being Index (PWBI) in the second phase.

For this aim, the Lifestyle Habits component was hypothesized to cause changes in Physical well-being, Autonomy, Financial resources, Diet and Physical activity, while the Social Context component was hypothesized to cause changes in Parent relations, Peers, School environment, and Bullying (analogous schemas were adopted for the social context (SC) and mental skills (MS) components).

Conceptually, the AVATAR methodology is based on a multidimensional construct, covering physical, emotional, mental, social, and cognitive components of well-being and functioning as perceived by adolescents (7).

**Figure 1:**
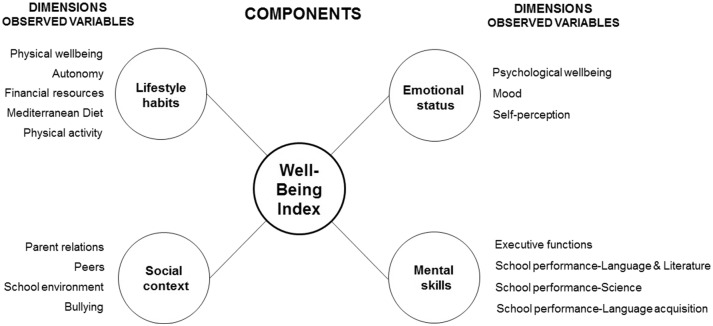
Conceptual framework for Well-Being Index (WBI).

The indicators belonging to the four components have been selected according to the analysis of the pre-existing literature in adolescents' health and well-being (15–17). Figure 1 depicts the single variables, namely observed variables, for each component.

### Statistical Analysis

All statistical analyses were completed using Stata/SE 13.1 and SPSS Version 24. Categorical variables were expressed as percentages, and continuous variables were expressed as mean and standard deviation (SD) and mean and standard error for standardized regression coefficient for the Structural Model. First, descriptive analyses were conducted to describe the sample. The level of significance was set at *p* < 0.05.

Structural equation modeling (SEM) by Stata/SE 13.1 was used to test the proposed model (Figure 1) (18, 19). The path analysis technique applied measures to the extent that the model fitted a data set and allowed testing of interrelationships between a range of variables simultaneously.

The SEM was used to test an overall measurement model that included four correlated latent variables. Overall model fit was assessed using different statistics. First, a chi-square analysis was used. The other indices were the Root Mean Square Error of Approximation (RMSEA) (values between 0.05 and 0.08 indicate acceptable fit, and values <0.05 a good fit), Comparative Fit Index (CFI) (values >0.90 indicate reasonable fit, >0.95 good fit), and Standardized Root Mean Square Residual (SRMR) (values <0.05 indicate good fit) (20). The measurement model was at first tested to ensure that each of the observed variables was a sufficient indicator of the hypothesized latent variables. Next, the model including the hypothesized pathways was evaluated.

### Personalised Well-Being Index

Data collected by the AVATAR Web-tool include information obtained by questionnaire. Both computed and user-provided data, obtained from the platform, can be mapped onto a specific axis of Σ space. Consequently, Personalised Well-Being Index (PWBI) implementation is referred to the estimation of the mappings between the predefined subareas (ΣL, ΣS, ΣE, and Σ MS) and the four axes (L, S, E, and MS):

Lifestyle habits (L): ΣL → ΩL

Social context (S): ΣS → ΩS

Emotional status (E): ΣE → ΩE

Mental skills (MS): → Σ MS Ω MS

Each of these causal relationships is modeled by a linear equation with the cause(s) as independent variable(s). Structural equation models (SEMs), widely used in psychometry and behavioral sciences, were used to implement the association phase (18). This choice was motivated by the moderate complexity of a linear SEM, which, despite a possible negative influence on the estimation accuracy, is advantageous with respect to overfitting issues.

In this view, estimation of the four components of the PWBI is based on the relation process illustrated in Figure 1 and PWBI was hypothesized to cause changes in Lifestyle habits (L), Social context (S), Emotional status (E), and Mental skills (MS).

Thanks to parameter estimates extracted from the SEM model (factor loadings and residual variance), an equation was defined to compute the (rescaled) PWBI of each student. Subsequently, in order to implement PWBI in the AVATAR web-tool for teachers and parents, the score PWBI was rescaled to a range of 0–100, using the following formula:

100^*^(PWBI - min)/(max - min).

The result of this equation was used as PWBI factor score for each student, a continuous trait that summarizes the common components of PWBI.

The equations are shown below.

PWBI = 0.954 ^*^ Lifestyle habits (L) + 0.972 ^*^ Social context (S) + 0.949 ^*^ Emotional status (E) + – 0.141 ^*^ Mental skills (MS).

## Results

### Measurement Model

As described in Supplementary Table 1, the four well-being components provided an acceptable explanation for their corresponding well-being dimensions, since all the coefficients were above 0.350 (21), with the exception of Diet and Physical Activity in LH component, and Executive Functions in the MS component, with a coefficient <0.350. Standardized regression coefficients, reported to the right in Supplementary Table 1, explain the contribution of each observed variables considered as predictor, to define the components. The model here reported can be overlapped on what was previously described in a sample of 756 adolescents (mean age 12.19, male 393) (22).

In the left part of Supplementary Table 1, the weights of the four components in the composition of the WBI in our sample are shown.

Thus, for WBI, the most important predictor was social context (β = 0.972, SE = 0.014, *p* < 0.000); subsequently, the WBI was strongly represented by lifestyle habits (β = 0.954, SE = 0.017, *p* < 0.000) and emotional status (β = 0.949, SE = 0.017, *p* < 0.000). Finally, mental skills (β = −0.1417, SE = 0.031, *p* < 0.0.00) resulted the least important predictor of WBI.

The standardized paths of the hypothesized health theory constructs from all the four components to their respective variables were specified in Figure 1. The Structural Model Fit indexes indicated that the proposed hypothetical model fitted the data (RMSEA = 0.074, SRMR was 0.039, and CFI = 0.910). The indexes for hypothetical model showed that the measurement model fitted adequately (23, 24).

### Personalised Well-Being Index

The factor scores of the selected SEM model were computed for each adolescent summing the original indicator variables for factor weights, so the Personalised (P) WBI algorithms were defined by the sum of each centered and scaled PWBI variable (x's) weighted by the corresponding ratio between factor loading (λ's) and residual variance (θ's):

$$\begin{array}{l} \text{PWBI}=\overset{p}{\underset{j=1}{\sum }}\frac{\lambda_{j}}{\theta_{j}}\times \left(\frac{x_{j}-\text{mean}\left(x_{j}\right)}{\text{sd}\left(x_{j}\right)}\right) \end{array}$$

**Figure 2:**
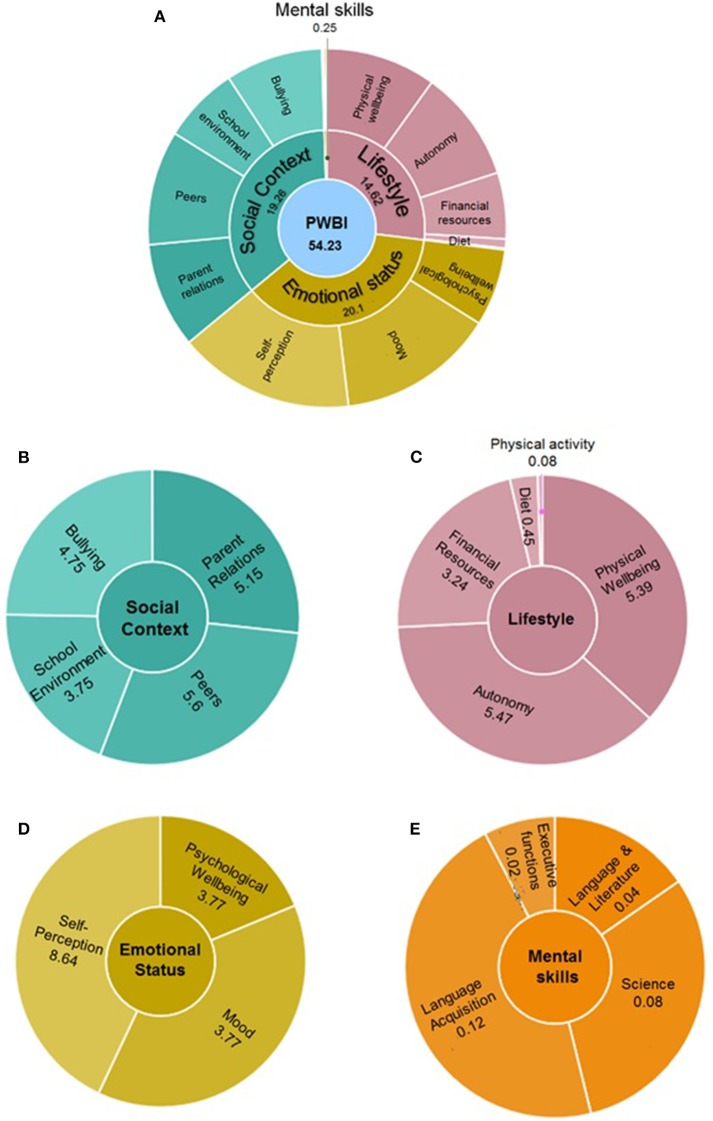
Graphical representation of the PWBI as it appears in the AVATAR web-tool. The global index (54.23) **(A)** is presented along with the social context **(B)**, lifestyle habits **(C)**, emotional status **(D)**, and mental skills **(E)** components.

Subsequently, the score PWBI was rescaled to 0–100 using 100^*^(PWBI e min)/(max e min). The result of this equation was adopted as the PWBI factor score for each individual, a continuous trait that summarizes the common components of PWBI. Figure 2 shows an example of graphical representation of the PWBI components in a student as they appear in the AVATAR platform. The PWBI offers personalised information, in accordance with the estimated PWBI in the total population, and its change over time. PWBI shows the different contribution of the four components and of the observed variables.

## Discussion

The present study showed the development of an innovative tool to assess well-being in healthy adolescents, in terms of Well-Being Index and in the second step as Personalised Well-Being Index based on the self-perception of well-being of individual adolescent. One important characteristic of this index is the integrated feature, due to the fact that the analysis is based on the relationship among the different weights of the variables belonging to the four dimensions: lifestyle, emotional status, social context, and mental skills. Furthermore, this index includes subjective information about health status based on the individual perception of health, that, according to WHO, corresponds to the “own position in life in the context of the culture and value systems in which subject lives and in relation to own goals, expectations, standards and concerns” (25, 26).

The main result of this study was the individuation of the social component (including parent relations, peers, school environment, and perception of bullying) as the most relevant in the definition of WBI, followed by lifestyle, emotional factors, and, lastly, mental skills.

This result is in line with evidences obtained from a study of school-aged children in which the World Health Organization underpins that social determinants are particularly important among adolescent aged 11–15 years, despite adolescents' health is typically measured by assessing rates of diseases or unhealthy behaviors, such as tobacco or substance abuse (27). Interestingly, the result of this study is in line with the evidence that changes linked to neurodevelopment in adolescence are essentially due to prominent structural and functional brain maturation related to continued synaptic pruning in specific neural systems, and this phenomenon is particularly observed in the cerebral areas involved in social processing (28, 29). Results obtained from neuroimaging, genetic, behavioral, and sociologic studies underline that the individual differences determined in adolescents' brain modulate the nature of social context, and, at the same time, social milieu exerts complex influences on adolescent behavior, and thus on well-being perception (29). Usually, social relationships, from family and peer-to-peer interactions, have traditionally been associated to risk-taking behaviors (30), emphasizing that, while the family plays a protective role (31), friends can promote the adoption of risk behaviors with deep consequences in adulthood (32). In fact, several studies in this field have suggested that changes in brain-based social sensitivity stimulate developmental paths that range from a successful transition to adulthood, and thus in resilience, to those culminating in psychopathology or maladaptation (17).

At the second level in the construction of WBI, there was the lifestyle component, with the leisure time having a higher weight also in comparison to diet and physical activity. This is an important result, since it is grafted onto the widespread concept that lifestyle habits improvement is mainly based on healthy and physical activity promotion. However, WBI index gains subjective information about health status of the adolescents and, therefore, gives additional information to the more objective evidences based on the beneficial effects of healthy diet and performance of physical activity (33, 34).

Emotional status component contributes to the definition of the PWBI at the third level, especially with psychological well-being variables, intended as positive emotions, satisfactions with life, and the absence of loneliness and sadness. Adolescence is a period associated with widespread changes in emotional behavior, with consequences on psychological and neurobiological development. In particular, the physiological reactivity to emotion, the capacity of emotional regulation, and the motivation to experiment with particular emotional states show complex trajectories that, if on the one hand develop the adolescent's resilience, on the other hand expose him or her to a greater vulnerability (35).

Lastly, mental skills contribute to create a well-being index, suggesting that this component is less important than the other psychosocial variables. Compared to the other components of more objective interpretation by the adolescents, awareness of mental skills requires a different mental processing that, consequently, changes their perception. Moreover, differently than the social component, which during the adolescence contributes to the creation of the individual's identity, the cognitive aspect, especially the executive function mediated by the prefrontal cortex, is still being defined and developed, thanks to the phenomenon of pruning (36).

### Practical Implications

PWBI is designed to achieve a Web-based health promotion tool for adolescents, parents, teachers, psychologists, and other stakeholders, with particular attention to school environment, considered as “context of socialization” that influences student's developmental outcomes (37). Importantly, this index allows to identify the strength and the fragile characteristics of each adolescent to potentiate the first ones and to change or improve the others through the application of personalized educational programs. In this context, PWBI intervenes at multiple points in the promotion process of health and well-being in the young people, combining management and empowerment in terms of ability to monitor well-being status, applying prevention strategies to reduce disease burden and health expenditure in adulthood. Indeed, PWBI may allow the adoption of more focused strategies and interventions to improve well-being. For example, on the bases of the different statistical weights of the four components and the different variables observed in the construction of the index, customized for each student, the teacher will be greatly supported to select more appropriately personalized interventions and educational programs and, thus, monitor over time their compliance and effectiveness. In this view and according AVATAR-PWBI, the schools have the opportunity to incorporate preventive approaches based on positive mental health, self-esteem, resilience, self-empowerment, and sociability into the educational curriculum in a non-threatening environment. According to the AVATAR approach to consider the school as a primary training ground for improving adolescent well-being, a Personal Well-Being Index-School Children (PWI-SC) has been previously validated in Portugal and Australia, and designed as a cross-cultural instrument to measure subjective well-being among high school-aged children (6). This index is mainly based on the subjective psychological assessment of mood happiness, in agreement with the key point that psychological well-being is a strong indicator of health.

### Limitations

Several limitations should be acknowledged. Firstly, given the reliance on self-report data, it would have been beneficial to conduct focus groups to explore concepts in more depth, although the use of self-report questionnaires allowed for the investigation in a large sample. Nonetheless, it is also important to collect directly by the interested subjects their experience and way of feeling, involving girls in first person. Secondly, since the questionnaires were completed during a school class, it is possible that the environment may have biased the students' responses. Third, the executive functions, at present measured only by the Stroop Color and Word Test Children's Version, must be enriched with other neurocognitive tests. Finally, the sample of adolescents cannot be considered diriment.

## Conclusions

The framework for PWBI developing here described shifts the traditional paradigm from the clinically disease-oriented to an educationally strength-based model in monitoring adolescents' psychosocial well-being. In light of the need for school-based health promotion and prevention initiatives, PWBI represents a significant opportunity for adolescents to feel empowered and learn life skills for health and well-being management, from an educational point of view. In addition, according to the American Heart Association statement on primordial prevention, claiming the development of comprehensive strategies, PWBI have potential implications on health, through psychosocial and environmental conditions' improvement, in order to prevent chronic degenerative diseases, in terms of morbidity and mortality, in the western countries (38). However, the PWBI is worth for further validation, and more studies are needed to show the potential benefits of this index to promote health and well-being in adolescents.

## Data Availability Statement

The datasets generated for this study will not be made publicly available because the data represents individuals under the age of 18. Requests to access the datasets should be directed to AP, pingi@ifc.cnr.it.

## Ethics Statement

The study was approved by the internal ethics committee of each participating School, in accordance with Italian law. Written informed consent to participate in this study was provided by the participants' legal guardian/next of kin.

## Author Contributions

FM, LB, CV, and AP contributed to the design of the study. GT, CD, and AC developed the web-platform. FM, LB, CV, and AP carried out the literature search, processed the review materials, and drafted the manuscript. FM, LB, GT, CD, AC, CV, and AP contributed to the data analyses and in writing the manuscript. All authors read and approved the final manuscript.

## Conflict of Interest

The authors declare that the research was conducted in the absence of any commercial or financial relationships that could be construed as a potential conflict of interest.
