# Supplementary material for: Adolescent Health: A Framework for Developing an Innovative Personalized Well-Being Index
Source: Front Pediatr. 2020 May 7;8:181. doi: 10.3389/fped.2020.00181 (PMC7223052; doi:10.3389/fped.2020.00181)
Supplement: Supplementary file 1 [file Table_1.doc]

**Table 1.** Standardized regression coefficient for the Structural Model in the WBI implementation

**PhyWB**: Physical Well-being; **AU**: Autonomy; **FR**: Financial resources; **DT**: Diet; **PA**: Physical Activity; **PR**: Parent Relations; **PE**: Peers; **SE**: School Environment; **BU**: Bullysm; **PsyWB**: Psychological Well-being; **MO**: Mood; **SP**: Self-Perception; **EM**: Emotion; **EF**: Executive Functions; **SPLL**: School performance-Language & Literature; **SPS**: School performance-Science; **SPLA**: School performance-Language Acquisition.
